# Supplementary material for: Product preferences and willingness to pay for potable water delivery: Experimental evidence from rural Bihar, India
Source: PLoS One. 2023 Apr 6;18(4):e0283892. doi: 10.1371/journal.pone.0283892 (PMC10079134; doi:10.1371/journal.pone.0283892)
Supplement: S1 File — (DOCX) [file pone.0283892.s001.docx]

**Appendix 1**

## **Auction 1:**

## **Willingness to pay auction script**

(to be enacted by the survey enumeration team)

## **Introduction:**

- We would like to sell you a one-week water delivery subscription, but the price is not yet fixed. It will be determined by chance in a game we are about to play.
- You will not have to spend any more for the water delivery subscription than you really want to.
- You may even be able to buy it for less. Here is how the promotion works:
  - I will ask you to tell me the maximum price you are willing to pay for the water delivery.
  - In this bag, I have many different pieces of paper with different numbers on them.
  - The numbers represent prices for the water delivery.
  - Then I will ask you to pick a piece of paper from the bag and we will look at the price together.
  - If the number you pick is less than or equal to your bid, you will buy the water delivery and you will pay the price you pick from the bag.
  - If the number you pick is greater than your bid, then you cannot buy the water delivery.
  - You will only have one chance to play for the water delivery.
  - You cannot change your bid after you draw from the bag.
  - You must state a price that you are actually able to pay now.
  - We will practice in one moment, but for now, do you have any questions?

***Practice round:***

Before we play for the water delivery, let’s practice the game. We’ll play the same game, but instead of playing for the water delivery, we will play for this bar of soap.

***Show respondent soap.***

1. What is the maximum amount that you are willing to pay for this soap?

***Respondent states price X, and enumerator records in survey software and on a separate sheet of paper for future reference***

| **WTP 3** | Initial bid | __ __ __ INR |
| --- | --- | --- |

1. Now, if you pick a number that is less than or equal to **X**, you will buy the soap at the price you pick. If you pick a number greater than **X**, you will not be able to purchase the soap, even if you are willing to pay the greater number. You cannot change your bid after you pick a price. Do you understand?
2. Please, tell me - if you pick [X+5 rupees] now, what happens?

***If respondent does not give correct answer, explain the rules again and then ask question again.***

1. And if you pick [X-5 rupees] now, what happens?

***If respondent does not give correct answer, explain the rules again and then ask question again.***

1. If you draw [X+5 rupees], will you want to purchase the soap for [X+5]?

***IF YES: Proceed to number* 6**
***IF NO: Skip to number 7***

1. Do you want to change your bid to [X+5]?

***IF YES:*** *“*OK, your new bid is [X+5].”
 ***Enumerator returns to number 2 and uses X+5 as the new X***
***IF NO:* *Proceed to number 7***

1. So, is ***X*** truly the most you would want to pay?

***IF NO:*** *“*What is the maximum amount that you are willing to pay for this soap?”
 ***Return to number 2, and use this new X*
*IF YES:*** ***Proceed to number 8***

1. If you pick ***X***, you must be able to pay ***X***. Are you able to pay ***X*** now?

***IF NO:*** *“*What is the maximum price you are willing and able to pay now?”
 ***Return to number 2, and use this new X
IF YES: Record respondent’s final bid in the software and proceed to 9***

| **WTP 4** | Final bid | __ __ __ INR |
| --- | --- | --- |

1. Could you please fetch the amount you have stated you are willing to pay and show it to me?

***Wait for respondent to fetch money and check to see she has enough funds for Final Bid.***

1. Now you will pick a price from the cup. If you pick X or less, you will buy the soap at the price you pick. If you pick more than X, you will not be able to buy the soap. Are you ready to pick a slip of paper?

***Mix slips of paper in a bag, hold bag above eye level of respondent and have him/her pick a slip of paper without looking.***

1. Now you can draw a slip of paper from the bag.

***Let respondent draw slip of paper. Together, look at the ball and read the price picked.*[*Drawn price is Y*] *Record Drawn Price***

| **WTP 5** | Price drawn | __ __ __ INR |
| --- | --- | --- |

1. et us look at the slip of paper together.

**[*If Y*** $\leq$ ***X*]:**

“The price is Y which is [less than/equal to] the amount you said you would be willing and able to pay for the soap. You can now buy the soap at this price.”
 ***Exchange payment for soap*.**

**[*If Y > X*]:**

“The price is Y, which is greater than the amount you said you would be willing to spend. You cannot purchase the soap.”

1. Do you have any questions about the game?

***Address any questions or concerns respondent has. Make sure he/she understands rules of game.***

***Sale round:***

- Now we will play to buy the water delivery subscription.
- Think back to the marketing information you were given by our colleague from the NGO.
  - **Remember that the price is a one-time payment for 7 days of water delivery and a bottle and dispenser.**
- Have you thought about how much you are willing to pay for the water delivery subscription?
- Do you have the funds available now?

Let’s begin:

1. What is the maximum amount that you are willing to pay for this water delivery subscription?

***Respondent states price X, and enumerator records in survey software and on a separate sheet of paper for future reference***

| **WTP 6** | Initial bid | __ __ __ INR |
| --- | --- | --- |

1. Now, if you pick a number that is less than or equal to ***X***, you will buy the water delivery subscription at the price you pick. If you pick a number greater than ***X***, you will not be able to purchase the water delivery, even if you are willing to pay the greater number. You cannot change your bid after you pick a price. Do you understand?
2. Please, tell me - if you pick [***X+*20 *rupees***] now, what happens?

***If respondent does not give correct answer, explain the rules again and then ask question again.***

1. And if you pick [***X-*20 *rupees***] now, what happens?

***If respondent does not give correct answer, explain the rules again and then ask question again.***

1. If you draw [***X+*20**], will you want to purchase the water delivery for [***X+*20**]?

***IF YES: Proceed to number* 6**
***IF NO: Skip to number 7***

1. Do you want to change your bid to [***X+*20**]?

***IF YES:*** *“*OK, your new bid is [***X+*20**].”
 ***Enumerator returns to number 2 and uses X+*20 *as the new X***
***IF NO:*** ***Proceed to number 7***

1. So, is ***X*** truly the most you would want to pay?

***IF NO:*** *“*What is the maximum amount that you are willing to pay for this water delivery subscription?”
 ***Return to number 2, and use this new X*
*IF YES:*** ***Proceed to number 8***

1. If you pick ***X***, you must be able to pay ***X***. Are you able to pay ***X*** now?

***IF NO:*** *“*What is the maximum price you are willing and able to pay now?”
 ***Return to number 2, and use this new X
IF YES: Record respondent’s final bid software and proceed to 9***

| **WTP 7** | Final bid | __ __ __ INR |
| --- | --- | --- |

1. Could you please fetch the amount you have stated you are willing to pay and show it to me?

***Wait for respondent to fetch money and check to see he/she has enough funds for Final Bid.***

1. Now you will pick a price from the bag. If you pick ***X*** or less, you will buy the water delivery at the price you pick. If you pick more than ***X***, you will not be able to buy the water delivery. Are you ready to pick a price?

***Mix slips of paper in bag, hold bag above eye level of respondent and have him/her pick a slip of paper without looking.***

1. Now you can draw a slip of paper from the bag.

***Let respondent draw slip of paper. Together, look at the ball and read the price picked.*[*Drawn price is Y*] *Record Drawn Price***

| **WTP 8** | Price drawn | __ __ __ INR |
| --- | --- | --- |

1. Let us look at the slip of paper together.

***Record if [Y] is less than or equal to or higher than [X] Final Bid Survey***

**[*If Y*** $\leq$ ***X*]:**

“The price is ***Y*** which is [less than/equal to] the amount you said you would be willing and able to pay for the water delivery subscription. You can now buy the water delivery subscription at this price.”
 ***Exchange payment for delivery subscription coupon*.**

**[*If Y > X*]:**

“The price is ***Y***, which is greater than the amount you said you would be willing to spend. You cannot purchase the water delivery subscription.”

## **Appendix 2**

## **Auction 2: Willingness to pay auction script**

(to be enacted by the survey enumeration team)

## **Introduction:**

- We would like to sell you a bottle and dispenser for a one-week water delivery subscription, but the price is not yet fixed. It will be determined by chance in a game we are about to play.
- You will not have to spend any more for the bottle, dispenser or water deliveries than you really want to.
- You may even be able to buy them for less. Here is how the promotion works:
  - First, I will ask you to draw a number from a bag to find out the price you will pay for one week of water delivery. The number will either be 5 (representing a 5 rupee per delivery price for seven deliveries for a total of 35 rupees) or 10 (representing a 10 rupees per delivery price for seven deliveries for a total of 70 rupees)
  - Next, I will ask you to tell me the maximum price you are willing to pay for the bottle and dispenser.
  - In a second bag, I have many different pieces of paper with different numbers on them.
  - The numbers represent prices for the bottle and dispenser.
  - Then I will ask you to pick a piece of paper from the second bag and we will look at the price together.
  - If the number you pick is less than or equal to your bid, you will buy the bottle and dispenser and you will pay the price you pick from the bag (plus the price of seven water deliveries you initially drew from the first bag – 35 or 70).
  - If the number you pick is greater than your bid, then you cannot buy the bottle and dispenser.
  - You will only have one chance to play for the bottle and dispenser to get the water deliveries.
  - You cannot change your bid after you draw from the bag.
  - You must state a price that you are actually able to pay now.
  - We will practice in one moment, but for now, do you have any questions?

***Practice round:***

Before we play for the bottle and dispenser, let’s practice the game. We’ll play the same game, but instead of playing for the bottle and dispenser, we will play for this bar of soap.

***Show respondent soap.***

1. What is the maximum amount that you are willing to pay for this soap?

***Respondent states price X, and enumerator records in survey software and on a separate sheet of paper for future reference***

| **WTP 3** | Initial bid | __ __ __ INR |
| --- | --- | --- |

1. Now, if you pick a number that is less than or equal to **X**, you will buy the soap at the price you pick. If you pick a number greater than **X**, you will not be able to purchase the soap, even if you are willing to pay the greater number. You cannot change your bid after you pick a price. Do you understand?
2. Please, tell me - if you pick [X+5 rupees] now, what happens?

***If respondent does not give correct answer, explain the rules again and then ask question again.***

1. And if you pick [X-5 rupees] now, what happens?

***If respondent does not give correct answer, explain the rules again and then ask question again.***

1. If you draw [X+5 rupees], will you want to purchase the soap for [X+5]?

***IF YES: Proceed to number* 6**
***IF NO: Skip to number 7***

1. Do you want to change your bid to [X+5]?

***IF YES:*** *“*OK, your new bid is [X+5].”
 ***Enumerator returns to number 2 and uses X+5 as the new X***
***IF NO:* *Proceed to number 7***

1. So, is ***X*** truly the most you would want to pay?

***IF NO:*** *“*What is the maximum amount that you are willing to pay for this soap?”
 ***Return to number 2, and use this new X*
*IF YES:*** ***Proceed to number 8***

1. If you pick ***X***, you must be able to pay ***X***. Are you able to pay ***X*** now?

***IF NO:*** *“*What is the maximum price you are willing and able to pay now?”
 ***Return to number 2, and use this new X
IF YES: Record respondent’s final bid in the software and proceed to 9***

| **WTP 4** | Final bid | __ __ __ INR |
| --- | --- | --- |

1. Could you please fetch the amount you have stated you are willing to pay and show it to me?

***Wait for respondent to fetch money and check to see she has enough funds for Final Bid.***

1. Now you will pick a price from the cup. If you pick X or less, you will buy the soap at the price you pick. If you pick more than X, you will not be able to buy the soap. Are you ready to pick a slip of paper?

***Mix slips of paper in a bag, hold bag above eye level of respondent and have him/her pick a slip of paper without looking.***

1. Now you can draw a slip of paper from the bag.

***Let respondent draw slip of paper. Together, look at the ball and read the price picked.*[*Drawn price is Y*] *Record Drawn Price***

| **WTP 5** | Price drawn | __ __ __ INR |
| --- | --- | --- |

1. Let us look at the slip of paper together.

**[*If Y*** $\leq$ ***X*]:**

“The price is Y which is [less than/equal to] the amount you said you would be willing and able to pay for the soap. You can now buy the soap at this price.”
 ***Exchange payment for soap*.**

**[*If Y > X*]:**

“The price is Y, which is greater than the amount you said you would be willing to spend. You cannot purchase the soap.”

1. Do you have any questions about the game?

***Address any questions or concerns respondent has. Make sure he/she understands rules of game.***

***Sale round:***

- First, I will ask you to draw a number from a bag.

***Respondent draws number from bag 1, and enumerator records the number drawn in survey software and on a separate sheet of paper for reference***

| **WTP 5a** | **Number Drawn** | **__ __ INR** |
| --- | --- | --- |

- Remember that this is the ADDITIONAL price that you will be obligated to pay for one week of water deliveries if you are successful in the game we are about to play for the bottle and dispenser. If you drew 5, you will be obligated to pay 35 rupees for seven water deliveries. If you drew 10, you will be obligated to pay 70 rupees for seven water deliveries.
- Now we will play to buy the bottle and dispenser.
- Think back to the marketing information you were given by our colleague from the NGO.
  - **Remember that the price is a one-time payment for a bottle and dispenser, and you will also pay [WTP 5a x 7] for 7 days of water delivery.**
- Have you thought about how much you are willing to pay for the bottle and dispenser?
- Do you have the funds available now?

Let’s begin:

1. What is the maximum amount that you are willing to pay for the bottle and dispenser?

***Respondent states price X, and enumerator records in survey software and on a separate sheet of paper for future reference***

| **WTP 6** | Initial bid | __ __ __ INR |
| --- | --- | --- |

1. Now, if you pick a number that is less than or equal to ***X***, you will buy the water delivery subscription at the price you pick. If you pick a number greater than ***X***, you will not be able to purchase the water delivery, even if you are willing to pay the greater number. You cannot change your bid after you pick a price. Do you understand?
2. Please, tell me - if you pick [***X+*20 *rupees***] now, what happens?

***If respondent does not give correct answer, explain the rules again and then ask question again.***

1. And if you pick [***X-*20 *rupees***] now, what happens?

***If respondent does not give correct answer, explain the rules again and then ask question again.***

1. If you draw [***X+*20**], will you want to purchase the water delivery for [***X+*20**]?

***IF YES: Proceed to number* 6**
***IF NO: Skip to number 7***

1. Do you want to change your bid to [***X+*20**]?

***IF YES:*** *“*OK, your new bid is [***X+*20**].”
 ***Enumerator returns to number 2 and uses X+*20 *as the new X***
***IF NO:*** ***Proceed to number 7***

1. So, is ***X*** truly the most you would want to pay?

***IF NO:*** *“*What is the maximum amount that you are willing to pay for this bottle and dispenser?”
 ***Return to number 2, and use this new X*
*IF YES:*** ***Proceed to number 8***

1. If you pick ***X***, you must be able to pay ***X***. Are you able to pay ***X*** now?

***IF NO:*** *“*What is the maximum price you are willing and able to pay now?”
 ***Return to number 2, and use this new X
IF YES: Record respondent’s final bid software and proceed to 9***

| **WTP 7** | Final bid | __ __ __ INR |
| --- | --- | --- |

1. Could you please fetch the amount you have stated you are willing to pay and show it to me?

***Wait for respondent to fetch money and check to see he/she has enough funds for Final Bid.***

1. Now you will pick a price from the bag. If you pick ***X*** or less, you will buy the bottle and dispenser at the price you pick. If you pick more than ***X***, you will not be able to buy the bottle and dispenser. Are you ready to pick a price?

***Mix slips of paper in bag, hold bag above eye level of respondent and have him/her pick a slip of paper without looking.***

1. Now you can draw a slip of paper from the bag.

***Let respondent draw slip of paper. Together, look at the ball and read the price picked.*[*Drawn price is Y*] *Record Drawn Price***

| **WTP 8** | Price drawn | __ __ __ INR |
| --- | --- | --- |

1. Let us look at the slip of paper together.

***Record if [Y] is less than or equal to or higher than [X] Final Bid Survey***

**[*If Y*** $\leq$ ***X*]:**

“The price is ***Y*** which is [less than/equal to] the amount you said you would be willing and able to pay for the bottle and dispenser. You can now buy the bottle and dispenser, and additionally pay **[X plus initial price drawn from hat in WTP 5a]** for a one-week water delivery subscription at this price.”
 ***Exchange payment for delivery subscription coupon and bottle and dispenser*.**

**[*If Y > X*]:**

“The price is ***Y***, which is greater than the amount you said you would be willing to spend. You cannot purchase the bottle and dispenser or the water delivery subscription.”

| **Appendix Table 1. Attribute balance frequency for discrete choice experiments** | | | |  |
| --- | --- | --- | --- | --- |
|  | Baseline DCE | | Endline DCE | |
|  | no | % | no | % |
|  |  |  |  |  |
| Price |  |  |  |  |
| Price ₹0 – “water that is free” | 119 | 24.5% | 134 | 27.9% |
| Price ₹3 – “water that costs ₹3” | 120 | 24.7% | 111 | 23.1% |
| Price ₹6 – “water that costs ₹6” | 107 | 22.0% | 126 | 26.3% |
| Price ₹9 – “water that costs ₹9” | 140 | 28.8% | 109 | 22.7% |
|  |  |  |  |  |
| Taste |  |  |  |  |
| (0) “Tastes like iron” | 220 | 45.3% | 218 | 45.4% |
| (1) “Tastes nice” | 266 | 54.7% | 262 | 54.6% |
|  |  |  |  |  |
| Convenience |  |  |  |  |
| (0) “Must be ordered for delivery” | 230 | 47.3% | 223 | 46.5% |
| (0) “You can get whenever you like” | 256 | 52.7% | 257 | 53.5% |
|  |  |  |  |  |
| Health |  |  |  |  |
| (0) “May not be safe to use” | 209 | 43.0% | 231 | 48.1% |
| (1) “Is safe to use” | 277 | 57.0% | 249 | 51.9% |
|  |  |  |  |  |
| Temperature |  |  |  |  |
| (0) “Is room temperature” | 247 | 50.8% | 228 | 47.5% |
| (1) “Is cold and refreshing” | 239 | 49.2% | 252 | 52.5% |
|  |  |  |  |  |
| Neighbors |  |  |  |  |
| (0) “Is NOT used by most all of my neighbors” | 238 | 49.0% | 224 | 46.7% |
| (1) “Is used by most all of my neighbors” | 248 | 51.0% | 256 | 53.3% |
|  |  |  |  |  |
| **Total choice sets presented** | **486** | | **480** | |

| **Appendix Table 2. Adjusted DCE, multivariate logistic regression (dy/dx) – All households** | | | | | | | | | |
| --- | --- | --- | --- | --- | --- | --- | --- | --- | --- |
|  | (1) | | | (2) | | | (3) | | |
|  | Baseline preferences | | | Endline preferences | | | Pooled analysis | | |
| VARIABLES | (dy/dx) | se | 95% CI | (dy/dx) | se | 95% CI | (dy/dx) | se | 95% CI |
| ₹0/delivery price | (reference) | | | (reference) | | | (reference) | | |
| ₹3/delivery price | -0.149** | (0.0671) | -0.2807, -0.0176 | -0.069 | (0.0606) | -0.1873, 0.0500 | -0.149** | (0.0696) | -0.2858, -0.0128 |
| ₹6/delivery price | -0.156** | (0.0611) | -0.2759, -0.0363 | -0.081 | (0.0556) | -0.1899, 0.0281 | -0.165** | (0.0673) | -0.2971, -0.0334 |
| ₹9/delivery price | -0.147** | (0.0602) | -0.2646, -0.0288 | -0.199*** | (0.0632) | -0.3232, -0.0756 | -0.136** | (0.0642) | -0.2617, -0.0101 |
| Taste | 0.017 | (0.0438) | -0.0688, 0.1030 | 0.100** | (0.0403) | 0.0214, 0.1793 | 0.000 | (0.0458) | -0.0897, 0.0899 |
| Convenience | -0.055 | (0.0457) | -0.1441, 0.0352 | -0.081* | (0.0462) | -0.1718, 0.0091 | -0.048 | (0.0472) | -0.1405, 0.0445 |
| Safety | 0.132*** | (0.0427) | 0.0488, 0.2160 | 0.114*** | (0.0439) | 0.0277, 0.1997 | 0.127*** | (0.0448) | 0.0388, 0.2143 |
| Temperature | 0.060 | (0.0406) | -0.0193, 0.1397 | -0.032 | (0.0436) | -0.1178, 0.0530 | 0.062 | (0.0422) | -0.0205, 0.1449 |
| Neighbors use | 0.038 | (0.0432) | -0.0466, 0.1228 | -0.018 | (0.0441) | -0.1045, 0.0686 | 0.045 | (0.0452) | -0.0435, 0.1335 |
| Time (0 = baseline, 1 = endline) | |  |  |  |  |  | -0.215** | (0.1067) | -0.4236, -0.0053 |
| CHARACTERISTIC*TIME | | |  |  |  |  |  |  |  |
| ₹0/delivery price * Time |  |  |  |  |  |  | (reference) | | |
| ₹3/delivery price * Time |  |  |  |  |  |  | 0.062 | (0.0859) | -0.1063, 0.2303 |
| ₹6/delivery price * Time |  |  |  |  |  |  | 0.078 | (0.0911) | -0.1008, 0.2561 |
| ₹9/delivery price * Time |  |  |  |  |  |  | -0.049 | (0.1056) | -0.2560, 0.1579 |
| Taste * Time |  |  |  |  |  |  | 0.088 | (0.0573) | -0.0245, 0.2002 |
| Convenience * Time |  |  |  |  |  |  | -0.040 | (0.0628) | -0.1626, 0.0836 |
| Safety * Time |  |  |  |  |  |  | -0.028 | (0.0621) | -0.1500, 0.0934 |
| Temperature * Time |  |  |  |  |  |  | -0.121* | (0.0635) | -0.2454, 0.0036 |
| Neighbors use * Time |  |  |  |  |  |  | -0.063 | (0.0646) | -0.1897, 0.0637 |
|  |  |  |  |  |  |  |  |  |  |
| Covariates | Yes | | | Yes | | | Yes | | |
| Neighborhood fixed effects | Yes | | | Yes | | | Yes | | |
|  |  |  |  |  |  |  |  |  |  |
| Pseudo R^2^ | 0.1311 | | | 0.1108 | | | 0.0895 | | |
| Household observations | 155 | | | 153 | | | 155 | | |
| Choice observations | 440 | | | 454 | | | 894 | | |
| Notes: Status Quo = 0, Alternative = 1; Alternative-specific constants are suppressed in the table above; Robustness check for Table 6; "(dy/dx)" denotes marginal effects; *** p<0.01, ** p<0.05, * p<0.1; Robust standard errors clustered at household-level; Comparison of characteristics to status quo scenario: "delivery price" compares to '₹0 delivery price' reference; "Taste" compares to a "Iron taste = 0"; "Convenience" compares to an "on-demand = 0" comparison; "Safety" compares to a "might cause sickness = 0" comparison; "Temperature" compares to a "cold = 0" comparison; "Neighbors use" compares to a "Neighbors use same source = 0" comparison; All three regressions control for dummies for auction type (combined auction, split auction (discount), split auction (no discount)), all covariates from Table 4, and neighborhood fixed effects. | | | | | | | | | |

| **Appendix Table 3. Adjusted DCE, multivariate logistic regression (dy/dx) – Customer households only** | | | | | | | | | |
| --- | --- | --- | --- | --- | --- | --- | --- | --- | --- |
|  | (1) | | | (2) | | | (3) | | |
|  | Baseline preferences | | | Endline preferences | | | Pooled analysis | | |
| VARIABLES | (dy/dx) | se | 95% CI | (dy/dx) | se | 95% CI | (dy/dx) | se | 95% CI |
| ₹0/delivery price | (reference) | | | (reference) | | | (reference) | | |
| ₹3/delivery price | -0.059 | (0.0906) | -0.2366, 0.1184 | 0.062 | (0.0859) | -0.1065, 0.2302 | -0.077 | (0.0869) | -0.2475, 0.0933 |
| ₹6/delivery price | 0.056 | (0.0588) | -0.0596, 0.1709 | -0.049 | (0.0906) | -0.2267, 0.1285 | 0.048 | (0.0588) | -0.0677, 0.1629 |
| ₹9/delivery price | -0.076 | (0.0738) | -0.2208, 0.0686 | -0.025 | (0.0768) | -0.1760, 0.1251 | -0.072 | (0.0761) | -0.2212, 0.0770 |
| Taste | 0.013 | (0.0556) | -0.0964, 0.1216 | 0.062 | (0.0580) | -0.0521, 0.1753 | 0.048 | (0.0539) | -0.0574, 0.1539 |
| Convenience | 0.079 | (0.0681) | -0.0541, 0.2129 | -0.014 | (0.0528) | -0.1177, 0.0893 | 0.082 | (0.0704) | -0.0556, 0.2203 |
| Safety | 0.112 | (0.0756) | -0.0359, 0.2607 | 0.068 | (0.0566) | -0.0431, 0.1789 | 0.113 | (0.0725) | -0.0292, 0.2551 |
| Temperature | -0.038 | (0.0509) | -0.1378, 0.0616 | 0.029 | (0.0577) | -0.0844, 0.1417 | -0.041 | (0.0556) | -0.1503, 0.0675 |
| Neighbors use | 0.046 | (0.0762) | -0.1038, 0.1948 | -0.049 | (0.0607) | -0.1681, 0.0698 | 0.061 | (0.0743) | -0.0848, 0.2063 |
| Time (0 = baseline, 1 = endline) | |  |  |  |  |  | -0.073 | (0.1265) | -0.3205, 0.1753 |
| CHARACTERISTIC*TIME | | |  |  |  |  |  |  |  |
| ₹0/delivery price * Time |  |  |  |  |  |  | (reference) | | |
| ₹3/delivery price * Time |  |  |  |  |  |  | 0.089 | (0.0818) | -0.0719, 0.2489 |
| ₹6/delivery price * Time |  |  |  |  |  |  | -0.122 | (0.1419) | -0.3997, 0.1565 |
| ₹9/delivery price * Time |  |  |  |  |  |  | 0.040 | (0.1099) | -0.1753, 0.2553 |
| Taste * Time |  |  |  |  |  |  | 0.019 | (0.0798) | -0.1375, 0.1754 |
| Convenience * Time |  |  |  |  |  |  | -0.073 | (0.0700) | -0.2103, 0.0640 |
| Safety * Time |  |  |  |  |  |  | -0.067 | (0.1041) | -0.2707, 0.1372 |
| Temperature * Time |  |  |  |  |  |  | 0.041 | (0.0734) | -0.1030, 0.1846 |
| Neighbors use * Time |  |  |  |  |  |  | -0.123 | (0.0993) | -0.3178, 0.0716 |
|  |  |  |  |  |  |  |  |  |  |
| Covariates | Yes | | | Yes | | | Yes | | |
| Neighborhood fixed effects | Yes | | | Yes | | | Yes | | |
|  |  |  |  |  |  |  |  |  |  |
| Pseudo R^2^ | 0.1829 | | | 0.2965 | | | 0.1830 | | |
| Household observations | 53 | | | 53 | | | 53 | | |
| Choice observations | 157 | | | 155 | | | 312 | | |
| Notes: Status Quo = 0, Alternative = 1; Alternative-specific constants are suppressed in the table above; Robustness check for Table 7; "(dy/dx)" denotes marginal effects; *** p<0.01, ** p<0.05, * p<0.1; Robust standard errors clustered at household-level; Comparison of characteristics to status quo scenario: "delivery price" compares to '₹0 delivery price' reference; "Taste" compares to a "Iron taste = 0"; "Convenience" compares to an "on-demand = 0" comparison; "Safety" compares to a "might cause sickness = 0" comparison; "Temperature" compares to a "cold = 0" comparison; "Neighbors use" compares to a "Neighbors use same source = 0" comparison; All three regressions control for all covariates from Table 4 (excluding auction type) and neighborhood fixed effects. | | | | | | | | | |

| **Appendix Table 4. Adjusted DCE, multivariate logistic regression (dy/dx) – Non-customer households only** | | | | | | | | | |
| --- | --- | --- | --- | --- | --- | --- | --- | --- | --- |
|  | (1) | | | (2) | | | (3) | | |
|  | Baseline preferences | | | Endline preferences | | | Pooled analysis | | |
| VARIABLES | (dy/dx) | se | 95% CI | (dy/dx) | se | 95% CI | (dy/dx) | se | 95% CI |
| ₹0/delivery price | (reference) | | | (reference) | | | (reference) | | |
| ₹3/delivery price | -0.207** | (0.0841) | -0.3720, -0.0423 | -0.097 | (0.0771) | -0.2480, 0.0543 | -0.191** | (0.0898) | -0.3669, -0.0150 |
| ₹6/delivery price | -0.302*** | (0.0844) | -0.4674, -0.1364 | -0.119* | (0.0718) | -0.2600, 0.0216 | -0.299*** | (0.0860) | -0.4671, -0.1299 |
| ₹9/delivery price | -0.222*** | (0.0820) | -0.3830, -0.0614 | -0.300*** | (0.0805) | -0.4581, -0.1425 | -0.179** | (0.0830) | -0.3418, -0.0166 |
| Taste | 0.051 | (0.0585) | -0.0636, 0.1658 | 0.111** | (0.0504) | 0.0123, 0.2098 | 0.021 | (0.0602) | -0.0969, 0.1389 |
| Convenience | -0.121** | (0.0609) | -0.2400, -0.0014 | -0.120** | (0.0579) | -0.2339, -0.0069 | -0.100 | (0.0627) | -0.2231, 0.0227 |
| Safety | 0.120** | (0.0577) | 0.0068, 0.2330 | 0.131** | (0.0593) | 0.0150, 0.2475 | 0.117** | (0.0583) | 0.0027, 0.2311 |
| Temperature | 0.098* | (0.0520) | -0.0039, 0.1999 | -0.003 | (0.0576) | -0.1156, 0.1103 | 0.103* | (0.0552) | -0.0054, 0.2109 |
| Neighbors use | 0.022 | (0.0598) | -0.0949, 0.1394 | 0.038 | (0.0572) | -0.0745, 0.1498 | 0.015 | (0.0600) | -0.1023, 0.1328 |
| Time (0 = baseline, 1 = endline) | |  |  |  |  |  | -0.223* | (0.1352) | -0.4880, 0.0421 |
| CHARACTERISTIC*TIME | | |  |  |  |  |  |  |  |
| ₹0/delivery price * Time |  |  |  |  |  |  | (reference) | | |
| ₹3/delivery price * Time |  |  |  |  |  |  | 0.066 | (0.1115) | -0.1525, 0.2844 |
| ₹6/delivery price * Time |  |  |  |  |  |  | 0.166 | (0.1082) | -0.0462, 0.3780 |
| ₹9/delivery price * Time |  |  |  |  |  |  | -0.098 | (0.1320) | -0.3567, 0.1606 |
| Taste * Time |  |  |  |  |  |  | 0.072 | (0.0728) | -0.0703, 0.2149 |
| Convenience * Time |  |  |  |  |  |  | -0.034 | (0.0878) | -0.2062, 0.1378 |
| Safety * Time |  |  |  |  |  |  | 0.002 | (0.0754) | -0.1459, 0.1496 |
| Temperature * Time |  |  |  |  |  |  | -0.139 | (0.0862) | -0.3081, 0.0296 |
| Neighbors use * Time |  |  |  |  |  |  | 0.024 | (0.0810) | -0.1350, 0.1825 |
|  |  |  |  |  |  |  |  |  |  |
| Covariates | Yes | | | Yes | | | Yes | | |
| Neighborhood fixed effects | Yes | | | Yes | | | Yes | | |
|  |  |  |  |  |  |  |  |  |  |
| Pseudo R^2^ | 0.1645 | | | 0.1085 | | | 0.0810 | | |
| Household observations | 100 | | | 100 | | | 100 | | |
| Choice observations | 277 | | | 299 | | | 576 | | |
| Notes: Status Quo = 0, Alternative = 1; Alternative-specific constants are suppressed in the table above; Robustness check for Table 8; "(dy/dx)" denotes marginal effects; *** p<0.01, ** p<0.05, * p<0.1; Robust standard errors clustered at household-level; Comparison of characteristics to status quo scenario: "delivery price" compares to '₹0 delivery price' reference; "Taste" compares to a "Iron taste = 0"; "Convenience" compares to an "on-demand = 0" comparison; "Safety" compares to a "might cause sickness = 0" comparison; "Temperature" compares to a "cold = 0" comparison; "Neighbors use" compares to a "Neighbors use same source = 0" comparison; All three regressions control for all covariates from Table 4 (excluding auction type) and neighborhood fixed effects. | | | | | | | | | |
