# Supplementary material for: Product preferences and willingness to pay for potable water delivery: Experimental evidence from rural Bihar, India
Source: PLoS One. 2023 Apr 6;18(4):e0283892. doi: 10.1371/journal.pone.0283892 (PMC10079134; doi:10.1371/journal.pone.0283892)
Supplement: Attachment — Submitted filename: Response to Reviewers.docx [file pone.0283892.s003.docx]

Reviewers' comments (16 January):

Reviewer's Responses to Questions

**Comments to the Author**

1. If the authors have adequately addressed your comments raised in a previous round of review and you feel that this manuscript is now acceptable for publication, you may indicate that here to bypass the “Comments to the Author” section, enter your conflict of interest statement in the “Confidential to Editor” section, and submit your "Accept" recommendation.

Reviewer #1: All comments have been addressed

Reviewer #2: (No Response)

2. Is the manuscript technically sound, and do the data support the conclusions?

The manuscript must describe a technically sound piece of scientific research with data that supports the conclusions. Experiments must have been conducted rigorously, with appropriate controls, replication, and sample sizes. The conclusions must be drawn appropriately based on the data presented.

Reviewer #1: (No Response)

Reviewer #2: Yes

3. Has the statistical analysis been performed appropriately and rigorously?

Reviewer #1: (No Response)

Reviewer #2: Yes

4. Have the authors made all data underlying the findings in their manuscript fully available?

The [PLOS Data policy](https://nam12.safelinks.protection.outlook.com/?url=http%3A%2F%2Fwww.plosone.org%2Fstatic%2Fpolicies.action%23sharing&data=05%7C01%7Cdrew.cameron%40yale.edu%7Cddcc920f40f04c9a627c08daf7f372df%7Cdd8cbebb21394df8b4114e3e87abeb5c%7C0%7C0%7C638094922248357479%7CUnknown%7CTWFpbGZsb3d8eyJWIjoiMC4wLjAwMDAiLCJQIjoiV2luMzIiLCJBTiI6Ik1haWwiLCJXVCI6Mn0%3D%7C3000%7C%7C%7C&sdata=r9K4RU4C4YmkScWuWUFKpWQrxWaP7ALgRGE3ElTF4kE%3D&reserved=0) requires authors to make all data underlying the findings described in their manuscript fully available without restriction, with rare exception (please refer to the Data Availability Statement in the manuscript PDF file). The data should be provided as part of the manuscript or its supporting information, or deposited to a public repository. For example, in addition to summary statistics, the data points behind means, medians and variance measures should be available. If there are restrictions on publicly sharing data—e.g. participant privacy or use of data from a third party—those must be specified.

Reviewer #1: (No Response)

Reviewer #2: Yes

5. Is the manuscript presented in an intelligible fashion and written in standard English?

PLOS ONE does not copyedit accepted manuscripts, so the language in submitted articles must be clear, correct, and unambiguous. Any typographical or grammatical errors should be corrected at revision, so please note any specific errors here.

Reviewer #1: (No Response)

Reviewer #2: Yes

6. Review Comments to the Author

Please use the space provided to explain your answers to the questions above. You may also include additional comments for the author, including concerns about dual publication, research ethics, or publication ethics. (Please upload your review as an attachment if it exceeds 20,000 characters)

Reviewer #1: I have read the responses to my earlier comments and the revised manuscript and am satisfied. As I said earlier, I truly appreciate the authors' transparency and commitment to high-quality and pre-registered research. I will now add that I also appreciate their willingness to take feedback so constructively.

We appreciated your constructive comments! Thank you for your careful reading of our manuscript.

Reviewer #2: Thanks to the authors for their responses and thorough revisions. I have just a few more comments, mostly minor.

Major comments:

In "2.3 Willingness to pay: Random price auctions" starting on line 253, how were households identified? This is important, as, if not random, using Auction 1 on the first 69 households and Auction 2 on the last 93 households can produce biased results.

Thank you for this comment. We have provided added clarity on pages 14-5 lines 301-11:

“Households were selected for the study during a listing process in which members of the study enumeration team walked through eligible neighborhoods and screened potential households for inclusion. Eligible households were not selected into Auction 1 versus Auction 2 by randomization, but rather by listing order, thus it is possible that household characteristics differ between the two groups, which could potentially introduce bias of uncertain sign in our comparisons of the auctions. Table 3 presents formal tests of differences in baseline characteristics between the two auction groups, and finds significant differences, indicating the importance of testing for sensitivity of results to controlling for these baseline characteristics so as to quantify potential bias at least from these observed differences. In Table 5 we present the main auction comparisons without versus with these baseline controls and find that adding these controls has only minor effects on the estimated coefficients and does not change our overall conclusions.”

Line 206 “Study Setting": How does this setting impact the interpretation of results? What population are we learning about?

We agree with the need for additional clarity here about the population under study. We have revised pages 9-10 lines 226-8 to read:
“Because this sample is slightly poorer than the average Bihari household, our results may have a slight downward bias with regard to product adoption, though they would likewise not be generalizable to the poorest of the poor households in the region.”

Paragraph starting on line 373: Even though you use a status-quo scenario and compare alternatives to this, with 124 possible scenarios but only 162 respondents, are you powered enough to find anything? And if so, is the N so small it may be biased?

Power is a potential concern for our study. Thus, we have updated the last sentence of the paragraph you highlighted to include the following on page 19 lines 405-13:

“Following Johnson et al. (2013), we eliminated all 'implausible' alternative product characteristic alternative scenarios that were strongly dominated by the status quo choice (e.g., “a delivered water product for a positive price that tastes bad, requires request for delivery, can make respondents sick, and is not used by neighbors”) leaving a total of 124 possible alternatives to the status quo to be presented to 162 respondents (3 times each, totaling 486 choices) over two rounds. Although this is a large number of alternatives relative to the sample size, the power implications of this are mitigated by following the standard methods in this literature of estimating only a small number of choice set parameters (eight). Our resulting confidence intervals confirm that we had sufficient power to estimate meaningful effect sizes.”

Minor comments:

Line 61: “Access to piped water has expanded significantly (Murray et al. 2020), but, as of 2020, just over 83% of urban households and 42% of rural households received piped water services (WHO/UNICEF 2021)”
Is this referring to the 2 billion households using contaminated water or all total households?

Thank you for pointing out the vague language. We have revised the passage on page 3 line 63 to read:

“Access to piped water has expanded significantly (Murray et al. 2020), but, as of 2020, just over 83% of urban households worldwide and 42% of rural households received piped water services (WHO/UNICEF 2021).”

Line 101-112 onward should be present tense. Same with line 137.

We have amended the relevant paragraphs, now pages 4-5 lines 101-110 and page 6 line 140, to read:

“This study has multiple aims designed to inform future WASH studies and programs on demand and preferences for clean water. The first is to examine willingness to pay (WTP) for a treated water delivery service among residents to assess product pricing both in real terms and as a share of household income to inform the literature on product pricing. Second, we seek to examine the possible role of subsidies in increasing short-term demand and product uptake to inform a larger, randomized controlled trial that examines the potential for subsidies to lead to price anchoring and/or positive learning. Third, we aim to identify specific product characteristics that are subject to experiential learning among new customers to add to existing knowledge on how preferences for water products change over time. In regions where public utilities are unlikely (for the present) to enter “last-mile” communities, a more complete understanding of the acceptability and affordability of treated and delivered water is essential for policymaking on safe drinking water solutions.”

And

“Among these studies, some of the most important water product characteristics are price, taste, smell, health benefits (both real and perceived), convenience of use (including time required), durability and aesthetics.”

Respectively.

Line 600: “though, notably, the coefficient on a price of ₹9 is large and negative.” What does this mean? What is the interpretation?

Since this result was insignificant, we have struck the line from the manuscript (see line 626).

Line 651 : “Our findings led to a failure to reject alternative hypothesis 2, suggesting that the effect of missing out on small discounts had modest but larger negative effects on demand.” How does this suggestion/interpretation depend on possible biases in estimates?

This is an important point, and one for which we include the following revised caveat on page 40 lines 680-83:

“Our findings led to a failure to reject alternative hypothesis 2, suggesting that the effect of missing out on small discounts had modest but larger negative effects on demand. We caution that there may be unobservable sources of bias inherent to those who were sampled for Auction 2 based on our non-random household listing procedure. However, the direction of potential bias is unclear.”

7. PLOS authors have the option to publish the peer review history of their article ([what does this mean?](https://nam12.safelinks.protection.outlook.com/?url=https%3A%2F%2Fjournals.plos.org%2Fplosone%2Fs%2Feditorial-and-peer-review-process%23loc-peer-review-history&data=05%7C01%7Cdrew.cameron%40yale.edu%7Cddcc920f40f04c9a627c08daf7f372df%7Cdd8cbebb21394df8b4114e3e87abeb5c%7C0%7C0%7C638094922248357479%7CUnknown%7CTWFpbGZsb3d8eyJWIjoiMC4wLjAwMDAiLCJQIjoiV2luMzIiLCJBTiI6Ik1haWwiLCJXVCI6Mn0%3D%7C3000%7C%7C%7C&sdata=CtH4rXOKogzLlQKYGmo82p%2BEcKmfiWcmbrWsMiGJRHU%3D&reserved=0)). If published, this will include your full peer review and any attached files.


If you choose “no”, your identity will remain anonymous but your review may still be made public.

**Do you want your identity to be public for this peer review?** For information about this choice, including consent withdrawal, please see our [Privacy Policy](https://nam12.safelinks.protection.outlook.com/?url=https%3A%2F%2Fwww.plos.org%2Fprivacy-policy&data=05%7C01%7Cdrew.cameron%40yale.edu%7Cddcc920f40f04c9a627c08daf7f372df%7Cdd8cbebb21394df8b4114e3e87abeb5c%7C0%7C0%7C638094922248357479%7CUnknown%7CTWFpbGZsb3d8eyJWIjoiMC4wLjAwMDAiLCJQIjoiV2luMzIiLCJBTiI6Ik1haWwiLCJXVCI6Mn0%3D%7C3000%7C%7C%7C&sdata=wHswIRTjMSyJiNPbUxyD7hRtF%2BBtbyaa%2FzN2WiLzfVA%3D&reserved=0).

Reviewer #1: **Yes:** Joseph Cook

Reviewer #2: No

[NOTE: If reviewer comments were submitted as an attachment file, they will be attached to this email and accessible via the submission site. Please log into your account, locate the manuscript record, and check for the action link "View Attachments". If this link does not appear, there are no attachment files.]

While revising your submission, please upload your figure files to the Preflight Analysis and Conversion Engine (PACE) digital diagnostic tool, [https://pacev2.apexcovantage.com/](https://nam12.safelinks.protection.outlook.com/?url=https%3A%2F%2Fpacev2.apexcovantage.com%2F&data=05%7C01%7Cdrew.cameron%40yale.edu%7Cddcc920f40f04c9a627c08daf7f372df%7Cdd8cbebb21394df8b4114e3e87abeb5c%7C0%7C0%7C638094922248357479%7CUnknown%7CTWFpbGZsb3d8eyJWIjoiMC4wLjAwMDAiLCJQIjoiV2luMzIiLCJBTiI6Ik1haWwiLCJXVCI6Mn0%3D%7C3000%7C%7C%7C&sdata=XdNvkUZP5H00wx4eKTGnnRvApQZJ2qD1AFz5Twaq%2F8U%3D&reserved=0). PACE helps ensure that figures meet PLOS requirements. To use PACE, you must first register as a user. Registration is free. Then, login and navigate to the UPLOAD tab, where you will find detailed instructions on how to use the tool. If you encounter any issues or have any questions when using PACE, please email PLOS at [figures@plos.org](mailto:figures@plos.org). Please note that Supporting Information files do not need this step.
